# Refractive Errors and Concomitant Strabismus: A Systematic Review and Meta-analysis

**DOI:** 10.1038/srep35177

**Published:** 2016-10-12

**Authors:** Shu Min Tang, Rachel Y. T. Chan, Shi Bin Lin, Shi Song Rong, Henry H. W. Lau, Winnie W. Y. Lau, Wilson W. K. Yip, Li Jia Chen, Simon T. C. Ko, Jason C. S. Yam

**Affiliations:** 1 Department of Ophthalmology and Visual Sciences, The Chinese University of Hong Kong, Hong Kong; 2 Joint Shantou International Eye Center, Shantou University and The Chinese University of Hong Kong, Shantou, China; 3 Department of Ophthalmology, Tung Wah Eastern Hospital, Hong Kong

## Abstract

This systematic review and meta-analysis is to evaluate the risk of development of concomitant strabismus due to refractive errors. Eligible studies published from 1946 to April 1, 2016 were identified from MEDLINE and EMBASE that evaluated any kinds of refractive errors (myopia, hyperopia, astigmatism and anisometropia) as an independent factor for concomitant exotropia and concomitant esotropia. Totally 5065 published records were retrieved for screening, 157 of them eligible for detailed evaluation. Finally 7 population-based studies involving 23,541 study subjects met our criteria for meta-analysis. The combined OR showed that myopia was a risk factor for exotropia (OR: 5.23, P = 0.0001). We found hyperopia had a dose-related effect for esotropia (OR for a spherical equivalent [SE] of 2–3 diopters [D]: 10.16, P = 0.01; OR for an SE of 3-4D: 17.83, P < 0.0001; OR for an SE of 4-5D: 41.01, P < 0.0001; OR for an SE of ≥5D: 162.68, P < 0.0001). Sensitivity analysis indicated our results were robust. Results of this study confirmed myopia as a risk for concomitant exotropia and identified a dose-related effect for hyperopia as a risk of concomitant esotropia.

Strabismus, a misalignment of both eyes, is a common ocular disorder in childhood populations. The prevalence estimates for concomitant strabismus ranged from 2.3% to 6.0% in children^1^,^2^. The consequences of strabismus can be devastating. First, it would lead to loss of binocularity and depth perception. It is also the most common cause of amblyopia, and as such contributes importantly to childhood visual impairment^3^,^4^. In particular, the long-term surgical successes for strabismus (such as intermittent exotropia) are not satisfactory, with only around 50% success 3-year after the operation^5^,^6^. In additional to these functional effects, it also has significant psychosocial consequences in terms of self-image^4^,^7^, negative social prejudice^8^,^9^ and even lower chance to get employed^10^.

The pathogenesis of different types of concomitant strabismus has not been well established. Many researchers have studied the association between accommodative esotropia and hyperopia^11^,^12^. However, the degree of increased risk of accommodative esotropia in relation to the severity of hyperopia is not well documented. On the other hand, the observed higher prevalence of concomitant exotropia in Asian^11^,^13^ than Caucasians has been postulated to be related to the high prevalence of myopia^14^,^15^, but this relationship has not been well substantiated. Furthermore, the association between other types of refractive errors (such as astigmatism and anisometropia) and different types of concomitant strabismus is not clear. Indeed, confirming these relationships is important, because it can provide insights into the pathophysiology of concomitant strabismus; as well as providing guideline on the managements of refractive error in the aspect of preventing strabismus development^14^. In this systematic review and meta-analysis, we aimed to evaluate the risks of development of different types of concomitant strabismus in relation to all types of refractive errors.

## Method

### Search Strategies

Literature search was performed via the Ovid platform in MEDLINE (available in the public domain at http://www.nlm.nih.gov/bsd/licensee/medpmmenu.html) and EMBASE (available in the public domain at http:www.embase.com/info/helpfiles/) database from their starting date to April 1, 2016. The Boolean logic was adopted in the search strategy. The following keywords were used as free words and also as MeSH terms: “strabismus”, “esotropia”, “exotropia”, “refractive errors”, “myopia”, “hyperopia” and “anisometropia”. Detailed search strategies were given in Supplementary Table 1. Reference lists of the eligible articles and reviews were manually screened for additional articles, if any, that had not been captured by the electronic search.

### Eligibility Criteria

The inclusion criteria for eligible studies were as follows: (1) population-based cross-sectional study; (2) the status of strabismus (heterotropia) was diagnosed by cover and uncover test; (3) refractive error of both eyes was documented; (4) the numbers or frequencies of strabismus and non-strabismus in each type of refractive error were reported, or odds ratio (OR) and 95% confidence interval (95% CI) of different refractive errors for strabismus were estimated. We excluded animal studies, case reports, reviews, abstracts, conference proceedings, editorials, non-English articles and reports with incomplete data.

### Data Extraction

All retrieved records were screened and reviewed by two independent reviewers (SMT and RYTC). Uncertainties were resolved by consensus with a third reviewer (JCSY). Data collected from each study included (1) study information including year of publication, country of study, age range of participants, definition of different refractive errors (including myopia, hyperopia, astigmatism and anisometropia), sample sizes; (2) numbers of strabismus in subjects with and without refractive errors, reported unadjusted and adjusted ORs and 95% CIs (or standard errors), and adjusted co-variables; and (3) numbers of esotropia and exotropia in subjects with and without refractive errors, if provided.

### Risk of Bias Assessment

All included studies were population-based cross-sectional studies. Therefore, the quality of studies was assessed via the modified Estabrooks’ Quality Assessment and Validity Tool^16^ by two reviewers (SMT and RYTC) independently, and disagreements were resolved through discussion with a third reviewer (JCSY). The modified Estabrooks’ tool contains 14 items, which were divided into two groups. Group I included probabilistic sample used, sample size appropriate for power, response rate exceeding 50%, validity, appropriate tests used, and CI reported. Group II included representative sample, sample drawn from multiple sites, cluster/stratified design, multiple adjusted, detective variable [primary outcome] directly measured/administrative, reliability, P values reported, and missing data managed appropriately. Study was considered to be of high risk when one item in Group I was marked as “No” or two items marked as “N/A”, or any two items from Group II marked as “No” or three items marked as “N/A”^16^.

### Data Analysis

We evaluated the association between concomitant strabismus and refractive errors by synthesizing the outcomes using meta-analysis. Among the eligible studies, four studies have classified concomitant strabismus into esotropia and exotropia^11^,^12^,^17^,^18^. We assessed the association of exotropia and esotropia with different kinds of refractive errors, i.e., myopia, hyperopia, astigmatism and anisometropia. The combined odds ratios (ORs) with 95% confidence intervals (CIs) of refractive errors as associated factors for exotropia and esotropia were analyzed. The Cochran Q statistic testing for heterogeneity across studies and the I^2^ statistic quantifying the proportion of total variation attributable to between-study heterogeneity were calculated^19^. The P value of Q statistics lower than 0.1 and I^2^ above 50% indicated high heterogeneity. If significant heterogeneity was detected, result from the random-effect model was adopted^20^, otherwise, the fixed-effect model was used^21^. Two articles have stratified hyperopia into different severities (SE of 2-3D, SE of 3-4D, SE of 4-5D and SE of > 5D), therefore ORs and 95% CIs of were combined to compare the risk of esotropia. Sensitivity analysis was performed to confirm the association by removing studies of higher risk of introducing bias. We also assessed the contribution of each study to the heterogeneity by sequentially omitting each study and recalculating the combined ORs. The Modified Egger’s regression test was used to assess the potential publication bias, where a P value less than 0.05 was considered statistically significant^22^. The Review Manager software (RevMan, version 5.2; the Nordic Cochrane Centre, The Cochrane Collaboration, Copenhagen; 2012) was used for data analysis. The Stata software (version 12; StataCorp LP, College Station, TX) was used to validate the results and perform the Egger’s test. P values less than 0.05 were considered statistical significant.

## Results

**Figure 1:**
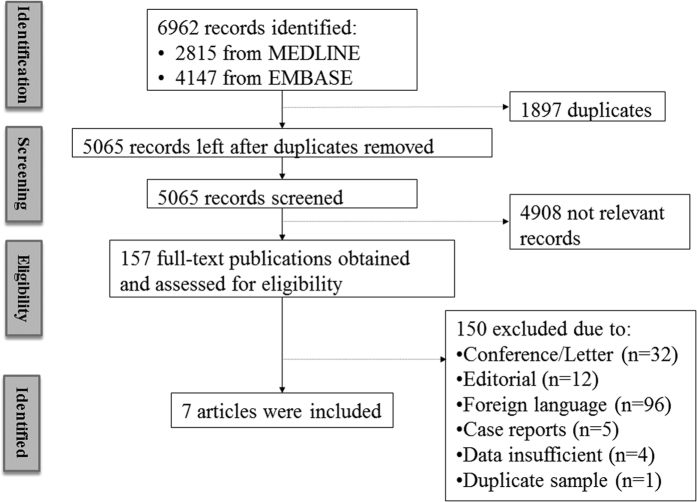
Flowchart of study inclusion.

From 1946 to April 1 of 2016, a total of 6962 publications were identified from the EMBASE and MEDLINE databases. After detailed screening and evaluation, 157 reports were eligible for detailed evaluation. Among them, we found 7 articles^1^,^11^,^12^,^17^,^18^,^23^,^24^ meeting our criteria for meta-analysis (Fig. 1). All these articles were population-based cross-sectional studies with the strabismus status and refractive error status of all included cases clearly documented. The studies spread across different ethnic groups, including Caucasian^11^,^12^,^17^,^23^ and East Asian^1^,^18^,^24^. Strabismus of all studies was evaluated using the cover-uncover test to define as any heterotropia at distant or near distance with or without spectacles. Cases of heterophoria were not eligible for the meta-analysis, and were excluded. Refractive errors, including myopia, hyperopia, astigmatism and anisometropia, were measured under cycloplegic condition in all included studies (Table 1). Overall, 23,541 subjects with age ranging from 6 months to 12 years were recruited for the meta-analysis in these 7 studies. Four articles^11^,^12^,^17^,^18^ have studied the association between refractive errors and different types of strabismus (including exotropia and esotropia). Three articles^12^,^17^,^18^ reported adjusted ORs and 95% CI for esotropia and exotropia, respectively. Factors that were usually adjusted included age, gender and refraction^12^,^17^,^18^ (Table 1).

### Association between myopia and concomitant strabismus

**Figure 2:**
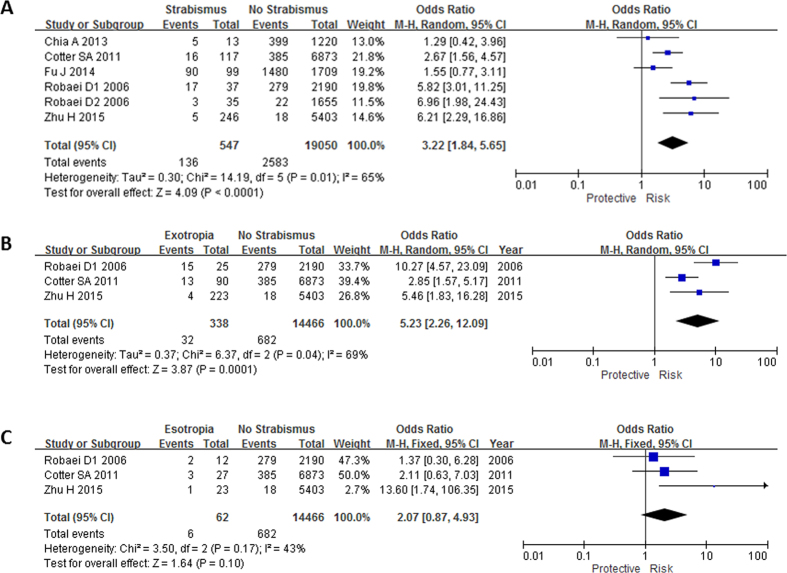
Meta-analysis of the association of myopia with different types of strabismus. The bars with squares in the middle represent 95% confidence intervals (95% CIs) and odds ratios (ORs). The central vertical solid line indicates the ORs for the null hypothesis. Diamond indicates summary OR with its corresponding 95% CI. (**A**) Association between myopia and strabismus. (**B**) Association between myopia and exotropia. (**C**) Association between myopia and esotropia.

Six out of seven studies^1^,^11^,^12^,^18^,^23^,^24^ have evaluated association between myopia and concomitant strabismus. Subjects with myopia had increased risk of developing concomitant strabismus (OR: 3.22, 95% CI: 1.84–5.65, I^2^ = 65%, P < 0.0001; Table 2, Fig. 2A). In subgroup analysis by strabismus subtypes pooled up by three studies^11^,^12^,^18^ (for myopia and hyperopia separately), myopia was associated with exotropia (OR: 5.23, 95% CI: 2.26–12.09, I^2^ = 69%, P = 0.0001; Table 3, Fig. 2B) but not associated with esotropia (OR: 2.07, 95% CI: 0.87–4.93, I^2^ = 43%, P = 0.1; Table 3, Fig. 2C). In the analysis using adjusted outcomes, myopia was associated with exotropia; but not associated with esotropia (OR: 2.63, 95% CI: 1.02–6.78, I^2^ = 0%, P = 0.05) (Table 4). Only Zhu’s paper^18^ has provided the adjusted OR for myopia and exotropia, which showed that myopia remained associated with exotropia after adjusting for age, gender and refraction.

### Association between hyperopia and concomitant strabismus

**Figure 3:**
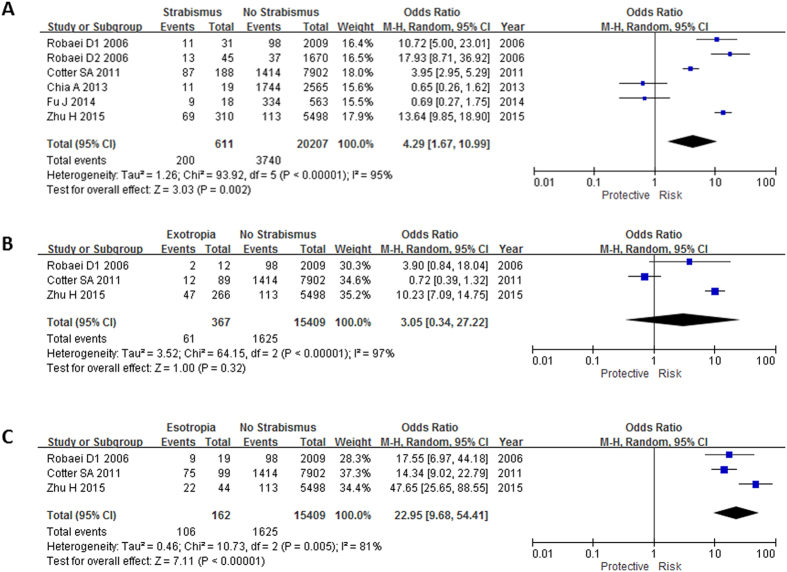
Meta-analysis of the association of hyperopia with different types of strabismus. The bars with squares in the middle represent 95% confidence intervals (95% CIs) and odds ratios (ORs). The central vertical solid line indicates the ORs for the null hypothesis. Diamond indicates summary OR with its corresponding 95% CI. (**A**) Association between hyperopia and strabismus. (**B**) Association between hyperopia and exotropia. (**C**) Association between hyperopia and esotropia.

Six out of seven studies^1^,^11^,^12^,^18^,^23^,^24^ have evaluated association between hyperopia and concomitant strabismus. Hyperopia was strongly associated with concomitant strabismus (OR: 4.29, 95% CI: 1.67–10.99, I^2^ = 95%, P = 0.002; Table 2, Fig. 3A). Three studies^11^,^12^,^18^ have evaluated association between hyperopia and esotropia; and exotropia separately. The subgroup analysis showed that hyperopia was a risk factor for esotropia (OR: 22.95, 95% CI: 9.68–54.41, I^2^ = 81%, P < 0.0001; Table 3, Fig. 3C), but not for exotropia (OR: 3.05, 95% CI: 0.34–27.22, I^2^ = 97%, P = 0.32; Table 3, Fig. 3B). Based on the severity of hyperopia, hyperopia was divided into 2-3D, 3-4D, 4-5D and higher than 5D. The risk of esotropia was increased with the severity of hyperopia (OR for a SE of 2-3D: 10.16; OR for a SE of 3-4D: 17.83; OR for a SE of 4-5D: 41.01; OR for a SE of ≥ 5D: 162.68; Table 5). Of note, after adjusted for age and gender, the association was still remained. (OR of SE = 2-3D: 7.26, OR of SE = 3-4D: 19.45, OR of SE = 4-5D: 44.86, OR of SE ≥ 5D: 134.19; Table 4).

### Association between astigmatism and concomitant strabismus

**Figure 4:**
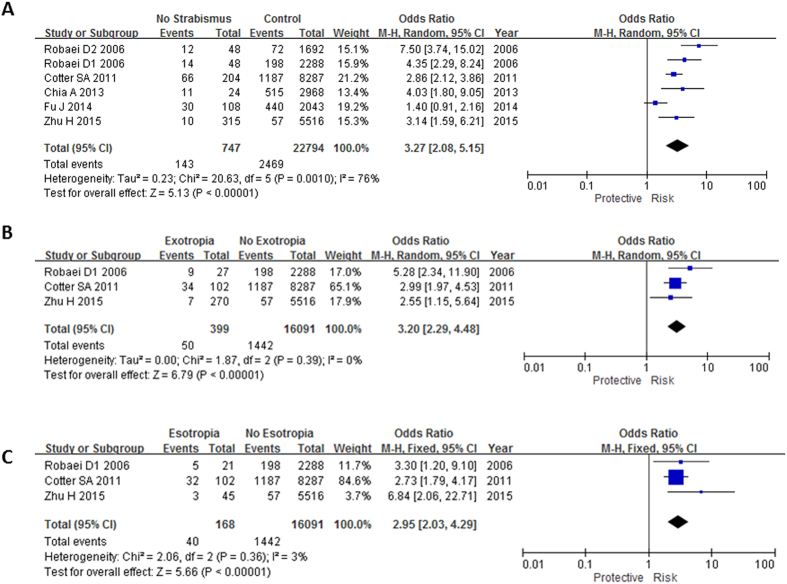
Meta-analysis of the association of astigmatism with different types of strabismus. The bars with squares in the middle represent 95% confidence intervals (95% CIs) and odds ratios (ORs). The central vertical solid line indicates the ORs for the null hypothesis. Diamond indicates summary OR with its corresponding 95% CI. (**A**) Association between astigmatism and strabismus. (**B**) Association between astigmatism and exotropia. (**C**) Association between astigmatism and esotropia.

Six out of seven studies^1^,^11^,^12^,^18^,^23^,^24^ have evaluated association between astigmatism and concomitant strabismus. Astigmatism was associated with concomitant strabismus (OR: 3.27, 95% CI: 2.08–5.15, I^2^ = 76%, P < 0.0001; Table 2, Fig. 4A). Three studies^11^,^12^,^18^ have evaluated association between astigmatism and exotropia; and esotropia separately. The subgroup analysis showed that astigmatism was associated with both the exotropia (OR: 3.20, 95% CI: 2.29–4.48, I^2^ = 0%, P < 0.0001; Table 3, Fig. 4B) and the esotropia (OR: 2.95, 95% CI: 2.03–4.29, I^2^ = 3%, P < 0.0001; Table 3, Fig. 4C). However, the associations of astigmatism with exotropia and esotropia became insignificant when using the adjusted ORs (P = 0.73; Table 4). Only Zhu’s paper^18^ has provided the adjusted OR for astigmatism and exotropia (OR: 1.06, 95% CI: 0.34–3.28, P = 0.919), in which the association could not withstand the adjustment.

### Association between anisometropia and concomitant strabismus

**Figure 5:**
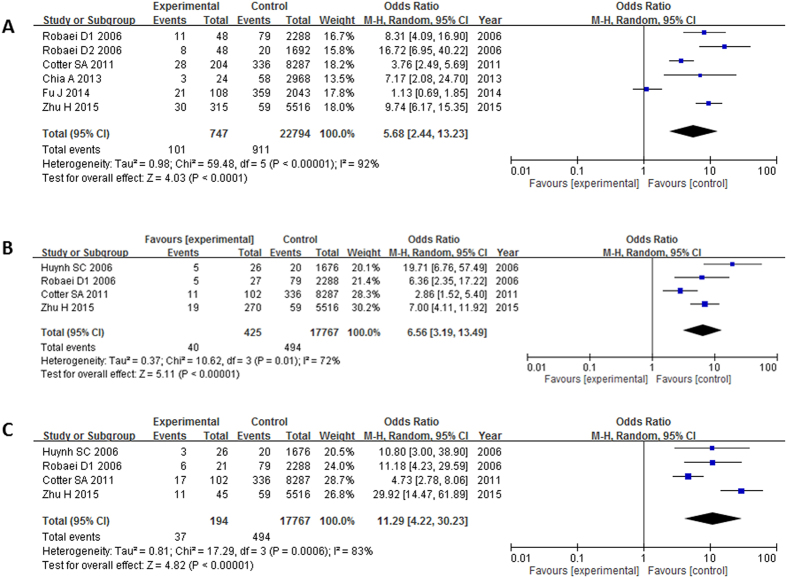
Meta-analysis of the association of anisometropia with different types of strabismus. The bars with squares in the middle represent 95% confidence intervals (95% CIs) and odds ratios (ORs). The central vertical solid line indicates the ORs for the null hypothesis. Diamond indicates summary OR with its corresponding 95% CI. (**A**) Association between anisometropia and strabismus. (**B**) Association between anisometropia and exotropia. (**C**) Association between anisometropia and esotropia.

Six out of seven studies^1^,^11^,^12^,^18^,^23^,^24^ have evaluated association between anisometropia and concomitant strabismus. Anisometropia was associated with concomitant strabismus (OR: 5.68, 95% CI: 2.44–13.23, I^2^ = 92%, P < 0.0001; Table 2, Fig. 5A). Four studies^11^,^12^,^17^,^18^ have evaluated association between anisometropia and exotropia; and esotropia separately. In subgroup analysis, anisometropia was a risk for both exotropia (OR: 6.56, 95% CI: 3.19–13.49, I^2^ = 72%, P < 0.0001; Table 3, Fig. 5B) and esotropia (OR: 11.29, 95% CI: 4.22–30.23, I^2^ = 83%, P < 0.0001; Table 3, Fig. 5C). However, the associations became insignificant after adjusting for confounding factors (P > 0.05; Table 4).

### Risk of bias assessment and sensitivity analysis

All studies were of high quality indicating low risk of bias when being included in this meta-analysis (Supplementary Table 2). Egger’s tests did not show significant findings in all of the analyses (Tables 2 and 3). Furthermore, we performed sensitivity analysis by omitting each study at a time subsequently to confirm the results. The association of hyperopia with exotropia became significant after removing Cotter’s study (OR: 9.59, 95% CI: 6.73–13.65, I^2^ = 31%, P < 0.0001)^12^. None of the other results was altered in the sensitivity analysis.

## Discussion

This systematic review and meta-analysis on 7 large-scale population-based studies involved 23,541 children to determine the association of refractive errors and concomitant strabismus. Based on the synthesized unadjusted ORs, all types of refractive errors (myopia, hyperopia, astigmatism and anisometropia) were confirmed as risk factors for concomitant strabismus.

Among the 7 studies, three have studied the association of myopia with exotropia and esotropia separately. Our results demonstrated no significant association between myopia and esotropia. On the other hand, children with myopia had 5.23-fold increase in risk to develop exotropia than those without significant ametropia. This may explain the high prevalence of exotropia in Asia, in which the prevalence of myopia is much higher^11^,^15^,^25^. The exact mechanism of how myopia may lead to exotropia is not certain. We postulated that the fusional control at distant of myopes is weakened due to the blurred distant vision. For near vision, less accommodative effort is required for clear image in myopes due to a larger accommodation lag^26^, which resulted in less accommodative convergence stimulated^27^. This prolonged suboptimal convergence may lead to breakdown of the fusional control and may predispose to exotropia development. In fact, previous studies have demonstrated that myopes without exotropia had a higher accommodative convergence to accommodation ratio (AC/A) than emmetropes, which also support our postulation^28^,^29^. The authors believed that myopes may require more convergence per accommodation in order to maintain good fusion and normal alignment, owing to the higher accommodation lag and thus less accommodative convergence stimulated. However, further prospective studies were warranted to confirm the postulation.

Our results revealed a very strong association between hyperopia and esotropia in a dose-related effect manner. We have therefore identified increasing risk of developing concomitant esotropia with the severity of hyperopia. Based on the meta-analysis of two papers^12^,^18^ involving around 12000 children, our results showed that hyperopia starting at the 2.00D to less than 3.00D imposes more than a 10-fold increase in risk of developing concomitant esotropia, and even up to 40-fold increase in risk for hyperopia up to 5.00D hyperopia. Strikingly, children with hyperopia of 5.00D or more had 218 times of risk of developing esotropia compared to children with 0.00D to less than 1.00D. This dose-related effect is highly relevant to public health. Health care providers should be cautioned in that children with moderate to high hyperopia should be closely monitored for the risk of developing into esotropia.

When pooling up the unadjusted ORs of three articles, which have separated into esotropia and exotropia, astigmatism was also found to be a risk for both esotropia and exotropia. However, the results need to be interpreted with cautions, because the confounding effect of spherical myopia and hyperopia has not been adjusted in the analysis. In the seven studies for this meta-analysis, only two reports have provided the adjusted ORs for the effects of astigmatism to exotropia. No association was found between astigmatism and exotropia based on the pooled adjusted ORs. Therefore, further studies were needed to confirm the association.

Anisometropia was found to be associated with strabismus and both esotropia and exotropia based on pooled unadjusted ORs. This can be attributed to the much reduced binocularity in children with anisometropia^30^. However, the pooled adjusted OR did not support the association of anisometropia with exotropia and esotropia. Therefore, there is still no definite conclusion on their relationship and further studies are required to confirm the association.

In this meta-analysis, all included studies were population-based studies. The results obtained are of epidemiological relevance. Furthermore, we used risk of bias assessment tools for observational study recommended by MOOSE guidelines and Cochrane handbook for Systematic Reviews, which showed all included studies were of good quality. Sensitivity analysis has been conducted to further confirm our findings and no significant publication bias has been found. However, all the 7 reports were cross-sectional studies. Further longitudinal studies are warranted to establish a causal relationship between the two conditions. Moreover, we found the definition of refractive errors varied among studies, which could be a major source of heterogeneities. In our meta-analysis, we accounted for heterogeneities by using random-effect model and sensitivity analysis.

In summary, we confirmed that myopia increased the risk of exotropia. Hyperopia was associated with increased risk of esotropia in a dose-dependent manner. We also reported a suggestive association of astigmatism and anisometropia with concomitant strabismus, which should be further confirmed in follow-up studies. Refractive errors are extremely common especially in Asian populations. Strabismus is a difficult ophthalmic disorder that disrupts vision and depreciates quality of life. Investigations are warranted to understand the pathophysiology for the associations between refractive errors and concomitant strabismus.

## Additional Information

**How to cite this article**: Tang, S. M. *et al.* Refractive Errors and Concomitant Strabismus: A Systematic Review and Meta-analysis. *Sci. Rep.* **6**, 35177; doi: 10.1038/srep35177 (2016).

## Supplementary Material

Supplementary Information

## Figures and Tables

**Table 1:** Summary of Included Studies.

First Author	Study-design	Location of Study	Sample size	Age	Strabismus	Definition					Adjusted factors for multivariate analysis	References
						Myopia	Hyperopia	Astigmatism	Anisometropia	Absent of significant ametropia		
Robaei D 2006	population-based cross-sectional	Sydney, AU	2352	12 y	Any heterotropia at near or distance fixation, or both.	SE ≤ −0.50 D	SE ≥ +2.00 D	cylinder ≥ 1.00 D	SE difference ≥ 1.00D	−0.50D < SE < +2.00D	N.A.	11
Robaei D 2006	population-based cross-sectional	Sydney, AU	1740	6 y	Any heterotropia at near (30 cm) or distance (6 m) fixation, or both.	SE ≤ −0.50 D	SE ≥ +2.00 D	cylinder ≥ 1.00 D	SE difference ≥ 1.00D	−0.50D < SE < +2.00D	clustering within school	23
Huynh SC 2006	population-based cross-sectional	Sydney, AU	1765	6 y	Any heterotropia at near (30 cm) or distance (6 m) fixation, or both.	N.A.	N.A.	N.A.	SE difference ≥ 1.00D	−0.50D < SE < +2.00D	Refraction, multiple birth and amblyopia	17
Cotter SA 2011	population-based cross-sectional	California and Maryland, USA	8491	6–72 m	Constant or intermittent heterotropia of any magnitude at distance or near fixation, or both.	SE ≤ −1.00 D	SE ≥ +2.00 D	cylinder ≥ 1.00 D	SE difference ≥ 1.00D	−1.00D < SE < +2.00D	gender, gestational age, age, maternal smoking during pregancy	12
Chia A 2013	population-based cross-sectional	Singapore	2992	6–72 m	Any manifest tropia identified on cover test.	SE ≤ −0.50 D	SE ≥ +0.50 D	cylinder ≥ 0.50 D	SE difference ≥ 1.00D	−0.50D < SE < +0.50D	age, gender, gestational age, admission to NICU, father’s education, sibling with strabismus, concurrent amblyopia	24
Fu J 2014	population-based cross-sectional	AnYang, China	2151	10–16 y	A heterotropia at near and/or distance fixation.	SE ≤ −0.50 D	SE ≥ +0.50 D	cylinder ≥ 1.00 D	SE difference ≥ 1.00D	−0.50D < SE < +0.50D	N.A.	1
Zhu H 2015	population-based cross-sectional	Nanjing, China	5831	3–6 y	Any tropia at distance or near, with or without spectacles	SE ≤ −1.00 D	SE ≥ +2.00 D	cylinder ≥ 1.00 D	SE difference ≥ 1.00D	−1.00D < SE < +2.00D	age and gender	18

**Table 2:** Meta-analysis of Association of Refractive Errors with Strabismus.

Type of exposure	No of Studies	Sample size	Overall effect			Heterogeneity		Egger’s	Reference
			OR (95%CI)	z score	P Value	I^2^,%	Q (P)		
Myopia	6	19597	3.22 (1.84–5.65)	4.09	<0.0001	65%	0.01	0.813	1,11,12,18,23,24
Hyperopia	6	20818	4.29 (1.67–10.99)	3.03	0.002	95%	<0.0001	0.541	1,11,12,18,23,24
Astigmatism	6	23541	3.27 (2.08–5.15)	5.13	<0.0001	76%	0.001	0.349	1,11,12,18,23,24
Anisometropia	6	23541	5.68 (2.44–13.23)	4.03	<0.0001	92%	<0.0001	0.505	1,11,12,18,23,24

**Table 3:** Meta-analysis of Association of Refractive Errors with Exotropia and Esotropia.

Type of exposure	No of Studies	Sample size	Overall effect			Heterogeneity		Egger’s	Reference
			OR (95%CI)	z score	P Value	I^2,^%	Q (P)		
**Association with exotropia**									
Myopia	3	14804	5.23 (2.26–12.09)	3.87	0.0001	69%	0.04	0.571	11,12,18
Hyperopia	3	15776	3.05 (0.34–27.22)	1	0.32	97%	<0.0001	0.682	11,12,18
Astigmatism	3	16490	3.2 (2.29–4.48)	6.79	<0.0001	0%	0.39	0.720	11,12,18
Anisometropia	4	18192	6.56 (3.19–13.49)	5.11	<0.0001	72%	0.01	0.951	11,12,17,18
**Association with esotropia**									
Myopia	3	14528	2.07 (0.87–4.93)	1.64	0.1	43%	0.17	0.454	11,12,18
Hyperopia	3	15571	22.95 (9.68–54.41)	7.11	<0.0001	81%	0.005	0.483	11,12,18
Astigmatism	3	16259	2.95 (2.03–4.29)	5.66	<0.0001	3%	0.36	0.344	11,12,18
Anisometropia	4	17961	11.29 (4.22–30.23)	4.82	<0.0001	83%	0.0006	0.585	11,12,17,18

**Table 4:** Adjusted ORs of the Association of Refractive Errors with Exotropia and Esotropia.

Type of exposure	No of Studies	Overall effect			Heterogeneity		Reference
		OR (95%CI)	z score	P Value	I^2,^%	Q (P)	
myopia vs esotropia	2	2.63 (1.02–6.78)	2	0.05	0%	0.77	12,18
hyperopia (2-3D) vs esotropia	2	7.26 (3.46–15.22)	5.25	<0.0001	0%	0.64	12,18
hyperopia (3-4D) vs esotropia	2	19.45 (8.79–43.02)	7.33	<0.0001	0%	0.38	12,18
hyperopia (4-5D) vs esotropia	2	44.86 (19.57–102.81)	8.99	<0.0001	45%	0.18	12,18
hyperopia (>5D) vs esotropia	2	134.19 (61.35–293.51)	12.27	<0.0001	0%	0.68	12,18
anisometropia vs esotropia	3	1.63 (0.32–8.27)	0.59	0.55	83%	0.003	12,17,18
astigmatism vs exotropia	2	1.63 (0.10–26.25)	0.34	0.73	86%	0.007	12,18
anisometropia vs exotropia	2	1.78 (0.14–22.87)	0.44	0.66	85%	0.009	12,18

**Table 5:** Association of Different Severity of Hyperopia with Esotropia.

Type of exposure	No of Studies	Sample size	Overall effect			Heterogeneity		Reference
			OR (95%CI)	z score	P Value	I^2,^%	Q (P)	
2-3D vs esotropia	2	12918	10.16 (1.58–65.38)	2.44	0.01	92%	0.0005	12,18
3-4D vs esotropia	2	12285	17.83 (10.17–31.25)	10.06	<0.0001	0%	0.5	12,18
4-5D vs esotropia	2	12062	41.01 (22.44–74.92)	12.08	<0.0001	0%	0.99	12,18
≥5D vs esotropia	2	12035	162.68 (40.91–646.89)	7.23	<0.0001	81%	0.02	12,18
