# Supplementary material for: Refractive Errors and Concomitant Strabismus: A Systematic Review and Meta-analysis
Source: Sci Rep. 2016 Oct 12;6:35177. doi: 10.1038/srep35177 (PMC5059633; doi:10.1038/srep35177)
Supplement: Supplementary Information [file srep35177-s1.pdf]

Title: Refractive Errors and Concomitant Strabismus: A Systematic Review and Meta-analysis

Author lists: Shu Min Tang, Rachel Y.T. Chan, Shi Bin Lin, Shi Song Rong, Henry H.W. Lau, Winnie W.Y. Lau, Wilson W.K. Yip, Li Jia Chen, Simon T.C. Ko, Jason C.S. Yam.

Supplementary table 1. Searching Strategy

1. exp refraction error/ or refraction.mp. or exp eye refraction/
2. refractive errors.mp.
3. exp myopia/ or myopia.mp. or exp high myopia/
4. anisometropia.mp. or exp anisometropia/ or exp aniseikonia/
5. aniseikonia.mp.
6. hypermetropia.mp. or exp hypermetropia/
7. hyperopia.mp.
8. 1 or 2 or 3 or 4 or 5 or 6 or 7
9. exp strabismus/ or strabismus.mp.
10. Esotropia.mp. or exp convergent strabismus/
11. Exotropia.mp. or exp divergent strabismus/
12. Cross-Eye.mp.
13. Esodeviation.mp.
14. Exodeviation.mp.
15. 9 or 10 or 11 or 12 or 13 or 14
16. 8 and 15

Supplementary table 2. Quality Assessment

First Author	Sample							Measurement			Statistical Analysis			
(year of publication)	Probabilistic sample used	Representative	Sample size appropriate for power	Sample drawn > 1 site	Cluster/stratified design	Multiple adjusted	Response rate > 50%	DV directly measured/administrative	DV reliability [c]	DV validity	Appropriate tests used	p values reported	CI reported	Missing data managed appropriately
Robaei D[1](2006)	Yes	Yes	Yes	Yes	Yes	N/A	Yes	Yes	N/A	Yes	Yes	Yes	Yes	N/A
Robaei D[2](2006)	Yes	Yes	Yes	Yes	Yes	Yes	Yes	Yes	N/A	Yes	Yes	Yes	Yes	N/A
Huynh SC (2006)	Yes	Yes	Yes	Yes	Yes	Yes	Yes	Yes	N/A	Yes	Yes	Yes	Yes	N/A
Cotter SA(2011)	Yes	Yes	Yes	Yes	Yes	Yes	Yes	Yes	N/A	Yes	Yes	Yes	Yes	N/A
Chia A(2013)	Yes	Yes	Yes	Yes	Yes	Yes	Yes	Yes	N/A	Yes	Yes	Yes	Yes	N/A
FU J (2014)	Yes	Yes	Yes	Yes	Yes	N/A	Yes	Yes	N/A	Yes	Yes	Yes	Yes	N/A
Zhu H(2015)	Yes	Yes	Yes	Yes	Yes	Yes	Yes	Yes	N/A	Yes	Yes	Yes	Yes	N/A
